# The role of endometrial thickness in predicting ectopic pregnancy after *in vitro* fertilization and the establishment of a prediction model

**DOI:** 10.3389/fendo.2022.895939

**Published:** 2022-09-08

**Authors:** Jing Liu, Hongjiao Kong, Xiaona Yu, Mengge Zhou, Xiaoyang Liu, Xinmi Liu, Jianrui Zhang, Yanli Liu, Shanshan Wu, Yichun Guan

**Affiliations:** ^1^ Reproductive Medicine Center, Third Affiliated Hospital of Zhengzhou University, Zhengzhou, China; ^2^ National Health Commission Key Laboratory of Birth Defects Prevention, Henan Institute of Reproduction Health Science and Technology, Zhengzhou, China

**Keywords:** IVF, ectopic pregnancy, risk factors, clinical prediction model, ROC

## Abstract

**Objective:**

To explore the risk factors of ectopic pregnancy after *in vitro* fertilization.

**Methods:**

This retrospective cohort study was conducted at the Reproductive Medical Center of the Third Affiliated Hospital of Zhengzhou University from January 2016 to April 2020. Univariate and multivariate analysis were used to analyze the related factors affecting the occurrence of ectopic pregnancy (EP) and to construct a nomographic prediction model for the incidence of ectopic pregnancy.

**Results:**

A total of 12,766 cycles of 10109 patients were included, comprising 214 cases of EP and 12,552 cases of intrauterine pregnancy (IUP). Multivariate logistic regression analysis showed that the tubal factor was associated with a 2-fold increased risk for EP (aOR = 2.72, 95% CI: 1.69-4.39, P < 0.0001). A stratified analysis showed that women with an endometrial thickness (EMT) between 7.6 to 12.1mm (aOR = 0.57, 95%CI: 0.36-0.90, P = 0.0153) and >12.1mm (aOR = 0.42, 95%CI: 0.24-0.74, P = 0.0026) had a significant reduction of the risk of EP compared to women with an EMT of <7.6mm. Compared to cleavage stage transfer, blastocyst transfer can reduce the risk of ectopic pregnancy (aOR = 0.36, 95%CI: 0.26-0.50, P < 0.0001). The saturation model (full mode) establishes a nomographic prediction model with an AUC = 0.68 and a sensitivity and specificity of 0.67and 0.64, respectively. The nomination model was internally verified by self-sampling method (bootstrap sampling resampling times = 500). The resulting AUC = 0.68 (sensitivity: 0.65; specificity: 0.65) showed that the model was relatively stable.

**Conclusions:**

Our findings indicate that EMT is inversely proportional to the risk of EP. Embryo stage, number of embryos transferred were also significantly associated with EP rate. A simple nomogram for the predicting the risk of EP was established in order to reduce the occurrence of EP.

## Introduction

The extrauterine implantation of an embryo is defined as ectopic pregnancy (EP) and is a serious complication of IVF (1). With the continuous development and extensive application of IVF over the last four decades, the prevalence of EP after IVF ranges between 2% to 11% (2), which is higher than 1% to 2% reported in natural pregnancies following spontaneous conceptions (3). Ectopic pregnancy leads to assisted pregnancy failure and at the same time increases the emotional and financial burden of a couple undergoing IVF treatment (4).

The mechanism of the occurrence of EP and reasons for increased risk of EP in IVF are not fully understood. Likewise, methods for reducing the incidence of EP are also limited. Therefore, the investigation of the high-risk factors that lead to EP following IVF and finding corresponding measures to decrease the risk of EP have attracted more and more attention by researchers. Previous studies suggested that the risk of EP may be related to the hyper-physiological hormone environment (5), tubal factor (6),embryonic factors (6), IVF procedure or other factors (7). The relationship between endometrial thickness (EMT) and the incidence of EP is still inconclusive. Some studies suggest that thin endometrium may be associated with the development of EP (8, 9). Similarly, Liu et al. found that the thickness of endometrium was negatively correlated with the incidence of EP during frozen embryo transfer cycles (10). However, a recent study did not find a correlation between endometrial thickness and EP (11). Still, we are lacking EP risk prediction models that are intuitive and can provide advice to patients at risk of EP.

The aim of this study was to evaluate factors that influence EP and to use the identified factors for establishing a potential risk assessment model for reduction of EP.

## Materials and methods

### Study design

**Figure 1:**
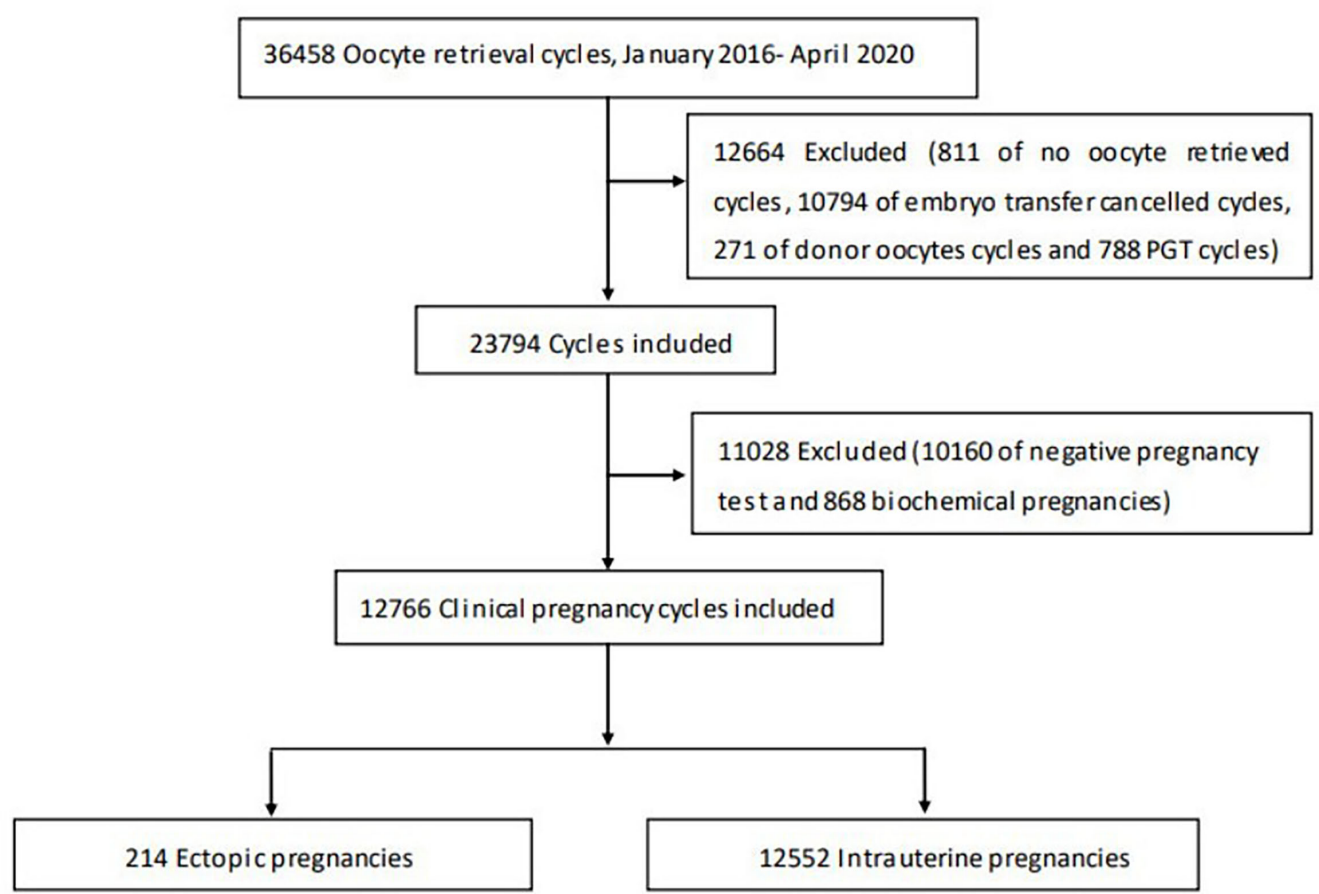
Flow chart of Study selection.

This retrospective cohort study was conducted at the Reproductive Medical Center of the Third Affiliated Hospital of Zhengzhou University. A total of 36,458 cycles that were performed between January 2016 to April 2020 were enrolled in the study. Cycles with incomplete clinical data, PGT, biochemical pregnancy and donor oocytes were excluded. A total of 12,766 IVF/ICSI-ET cycles performed at our reproductive center were finally included, 5,791 fresh cycles and 6,761 FET cycles. A flow-chart illustrating the identification and selection of cycles is presented in **Figure 1**.

### Clinical and laboratory protocols and embryo quality assessment

Conventional gonadotropin-releasing hormone agonist (GnRH-a) long protocol was used for ovarian stimulation, and a long-acting GnRH agonist (Dophereline, Ipsen Pharma Biotech, France) was used for down-regulation on the second to the fourth day of the menstrual cycle or the middle of the luteal phase. When the standard of pituitary down-regulation was reached (Luteinizing hormone (LH) < 5 IU/L, estradiol < 50 ng/L, endometrial thickness < 5 mm, no functional ovarian cyst), gonadotropins (gonadotropin, Gn, Merck Serono) were given for ovarian stimulation. The dosage of Gn was adjusted according to ovarian reactivity and hormone levels during medication.

All ultrasound examinations were performed by two highly trained sonographers using the GE E8 ultrasound system with its three-dimensional vaginal ultrasound probe; the median sagittal section of the uterus was obtained and the thickness of the double layer endometrium was measured at about 10mm from the uterine fundus, with the average value taken after several measurements. When the mean diameter of the dominant follicle was ≥ 20 mm or three follicles presented with a diameter ≥ 18 mm, hCG (Aizer, Merck, Germany) was administered. After 36~38 hours, oocyte retrieval was performed by transvaginal ultrasound guidance. Luteal support was routinely done after oocyte retrieval. According to the patient’s condition, fertilization was performed using either *in vitro* fertilization (IVF) or intracytoplasmic sperm injection (ICSI). Embryos were cultured at 6% CO_2_, 5% O_2_, 37°C in G-1™ Plus (Vitrolife, Sweden) from the pronuclear stage to cleavage stage, followed by G-2™ Plus (Vitrolife, Sweden) from cleavage stage to the blastocyst stage.

### Embryo quality assessment

Quality of cleavage stage embryos was assessed on day 3 and defined as:

Grade I with 6 to 10 cells and less than 5% fragmentation, Grade II with 6 to 10 cells and 5-20% fragmentation, Grade III embryos with uneven cell size, irregular cell morphology, and fragmentation from 21% to 50%. Grade IV embryos were defined as those with extremely uneven cell size, a large number of intracellular vacuoles, arrest of embryonic development or more than 50% embryo fragmentation. Grade I and II embryos were defined as good-quality embryos and Grade III embryos as poor-quality embryos. Grade I/II/III embryos were considered as utilizable embryos whereas grade IV embryos were defined as non-utilizable.

Blastocyst quality was assessed according to the scoring system of Gardner (12), based on blastocyst expansion and hatching, inner cell mass (ICM) and trophectoderm (TE) classification. According to the policy of our center, embryos graded ≥ 4BB (AA, AB, BA, BB)were considered as good blastocysts that were utilized for freezing and transplantation. All non-high-quality blastocysts (< 4BB, AC, BC) were defined as non-usable.

### Definition of tubal factors, EP and intrauterine pregnancy (IUP)

Infertility caused by fallopian tube factors accounts for about 25-35% (13). The diagnosis mainly depends on salpingography, laparoscopy and hysteroscopy (13, 14). The cycles of fallopian tube obstruction, hydrosalpinx, previous salpingectomy and salpingitis diagnosed by the above auxiliary examination or operation were included in this study.

For all patients hCG testing was performed 14 days after embryo transfer. When the serum hCG was >50 IU/L, transvaginal ultrasound was performed 29 days after embryo transfer. Clinical pregnancy was defined as the presence of a gestational sac. The implantation of at least one embryo in the uterus was defined as IUP. Ectopic pregnancy was defined as the presence of at least one gestational sac outside the uterine cavity. The simultaneous occurrence of an intra- and extra-uterine gestational sac was also classified as EP.

This study was approved by the Institutional Review Board of our hospital, which does not require signing the relevant informed consent form. The date of approval by the ethics committee is October 28, 2020, and the approval number is 2020-98.

### Statistical analysis and analyzed variables

The descriptive data of population characteristics were summarized using the mean ± standard for continuous variables. Counts and proportions were used for the categorical variables. Categorical variables were evaluated by the Chi-square (χ^2^) tests and Fisher’s exact tests. The t-test (normally distributed variables) or the Kruskal–Wallis test (abnormally distributed variables) were used to compare the continuous variables. Univariate and multivariate logistic regression analyses were performed to analyze various factors affecting EP. Smooth curve fitting was performed to examine whether the EMT is partitioned into intervals. If a non-linear relationship was observed, a three-piecewise linear regression model was constructed to calculate the threshold effect of EMT on EP rate according to the resultant smoothing plot. A recursive method was then employed to automatically determine the inflection point to be used in subsequent analyses (15). The full mode model was used to establish the line prediction model, the bootstrap sampling method was used to conduct internal verification of the line prediction model, and the area under the ROC curve (AUC) was used to evaluate the prediction performance of the line prediction model. All analysis was done using Empower (R) (www.empowerstats.com, X&Y solutions, inc. Boston MA) and R (http://www.R-project.org). P values less than 0.05 (two sided) were considered statistically significant.

## Results

The baseline clinical characteristics of the two groups (IUP vs. EP) are presented in **Table 1**. A total of 12,766 cycles of 10109 patients were screened for clinical data who met the inclusion criteria of this study, including 12,552 IUP and 214 EP, with an incidence of EP of 1.7%. The EMT in the IUP group was higher than that in the EP group (10.33 ± 2.16 vs 9.72 ± 2.18; P<0.001). The proportion of tubal factor infertility in the EP group was higher than in the IUP group (46.73% vs 37.75%; P = 0.007). Patients with a tubal factor were more likely to present with an EP than those without a tubal factor (2.11% vs 1.46%; P = 0.007). In EP group, the ratio of embryo transferred at cleavage stage (75.23% vs 53.11%; P<0.001) and double embryo transfer (75.23% vs 61.71%; P<0.001) are both higher than those in the IUP group. There was no statistical difference for any of the other basic data between the two groups (P > 0.05).

**Table 1:** Baseline characteristics of the patients.

	IUP (n = 12552)	EP (n = 214)	P-value
Female age (years)	30.77 ± 4.61	31.10 ± 4.77	0.299
Infertility duration (years)	3.41 ± 2.75	3.64 ± 3.20	0.232
Endometrial thickness prior to ET (mm)	10.33 ± 2.16	9.72 ± 2.18	< 0.001
BMI (kg/m2)	23.59 ± 3.23	23.48 ± 3.21	0.633
Infertility type			0.114
primary	5844 (46.56%)	88 (41.12%)	
secondary	6708 (53.44%)	126 (58.88%)	
Year of treatment			0.56
2016	2381 (18.97%)	39 (18.22%)	
2017	2860 (22.79%)	41 (19.16%)	
2018	3193 (25.44%)	64 (29.91%)	
2019	3730 (29.72%)	64 (29.91%)	
2020	388 (3.09%)	6 (2.80%)	
Caesarean section			0.324
NO	11918 (94.95%)	200 (93.46%)	
YES	634 (5.05%)	14 (6.54%)	
Previous EP, n (%)			0.452
NO	12438 (99.09%)	211 (98.60%)	
YES	114 (0.91%)	3 (1.40%)	
Curettage of the uterine cavity			0.467
NO	12521 (99.75%)	214 (100.00%)	
YES	31 (0.25%)	0 (0.00%)	
Type of infertility			0.007
Non-tubal factor	7813 (62.25%)	114 (53.27%)	
Tubal factor	4739 (37.75%)	100 (46.73%)	
Fresh or frozen embryo transfer			0.52
Fresh	5791 (46.14%)	94 (43.93%)	
Frozen	6761 (53.86%)	120 (56.07%)	
Embryo stage at ET			<0.001
cleavage stage	6666 (53.11%)	161 (75.23%)	
blastocyst stage	5886 (46.89%)	53 (24.77%)	
Embryo quality			0.523
Poor	5766 (45.94%)	103 (48.13%)	
Good	6786 (54.06%)	111 (51.87%)	
No. of embryos transferred			<0.001
single embryo transfer	4806 (38.29%)	53 (24.77%)	
double embryo transfer	7746 (61.71%)	161 (75.23%)	

EP, ectopic pregnancy; IUP, BMI, body mass index.

### Association between EMT and ectopic pregnancy

**Figure 2:**
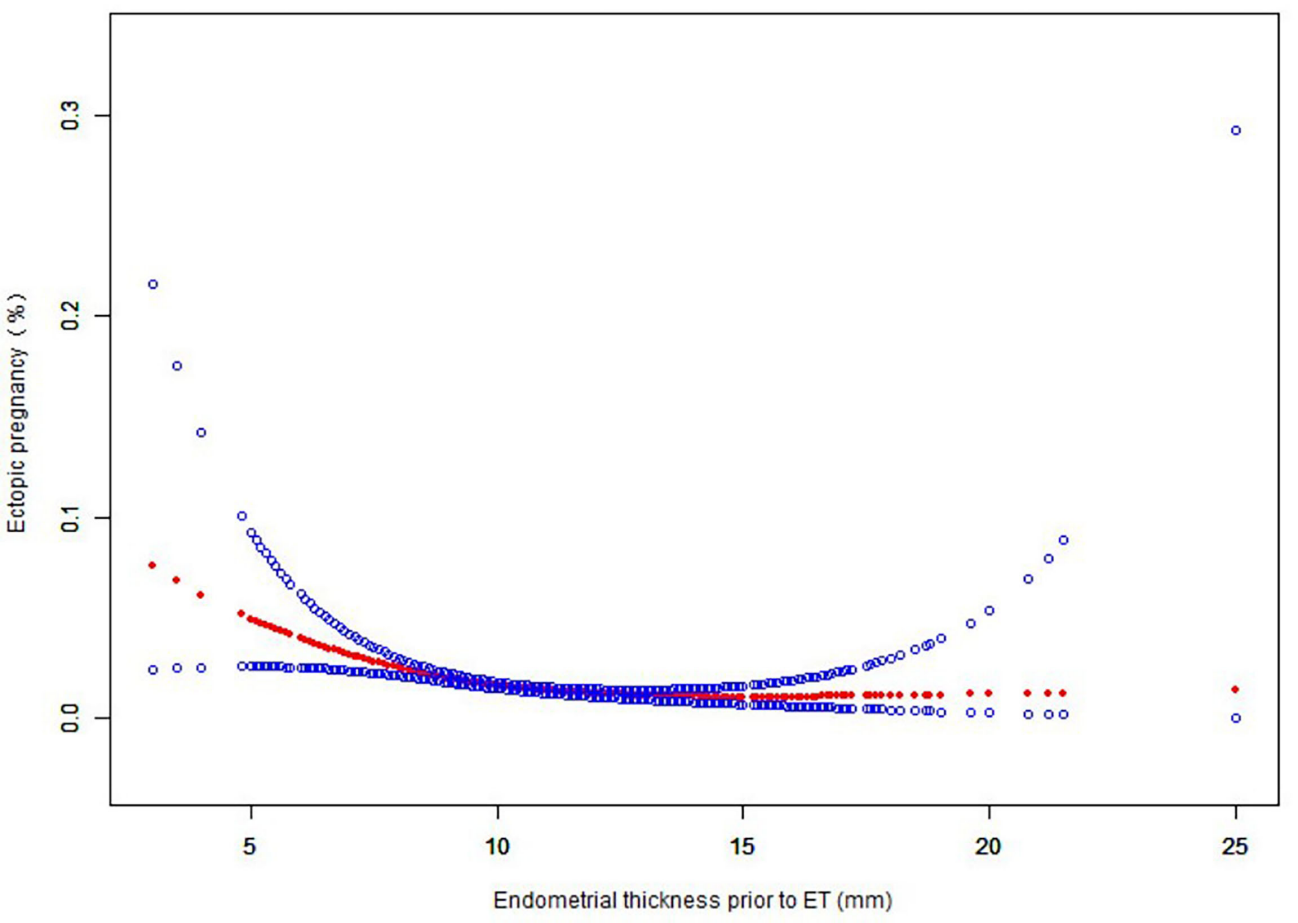
Nonlinear relationship between EMT and EP. *Adjusted factors: Female age, Infertility duration, BMI, Infertility type, Year of treatment, Cesarean section, Previous EP, Curettage of the uterine cavity, Type of infertility, Fresh or frozen embryo transfer, Embryo stage at ET, Embryo quality, No. of embryos transferred.

**Figure 3:**
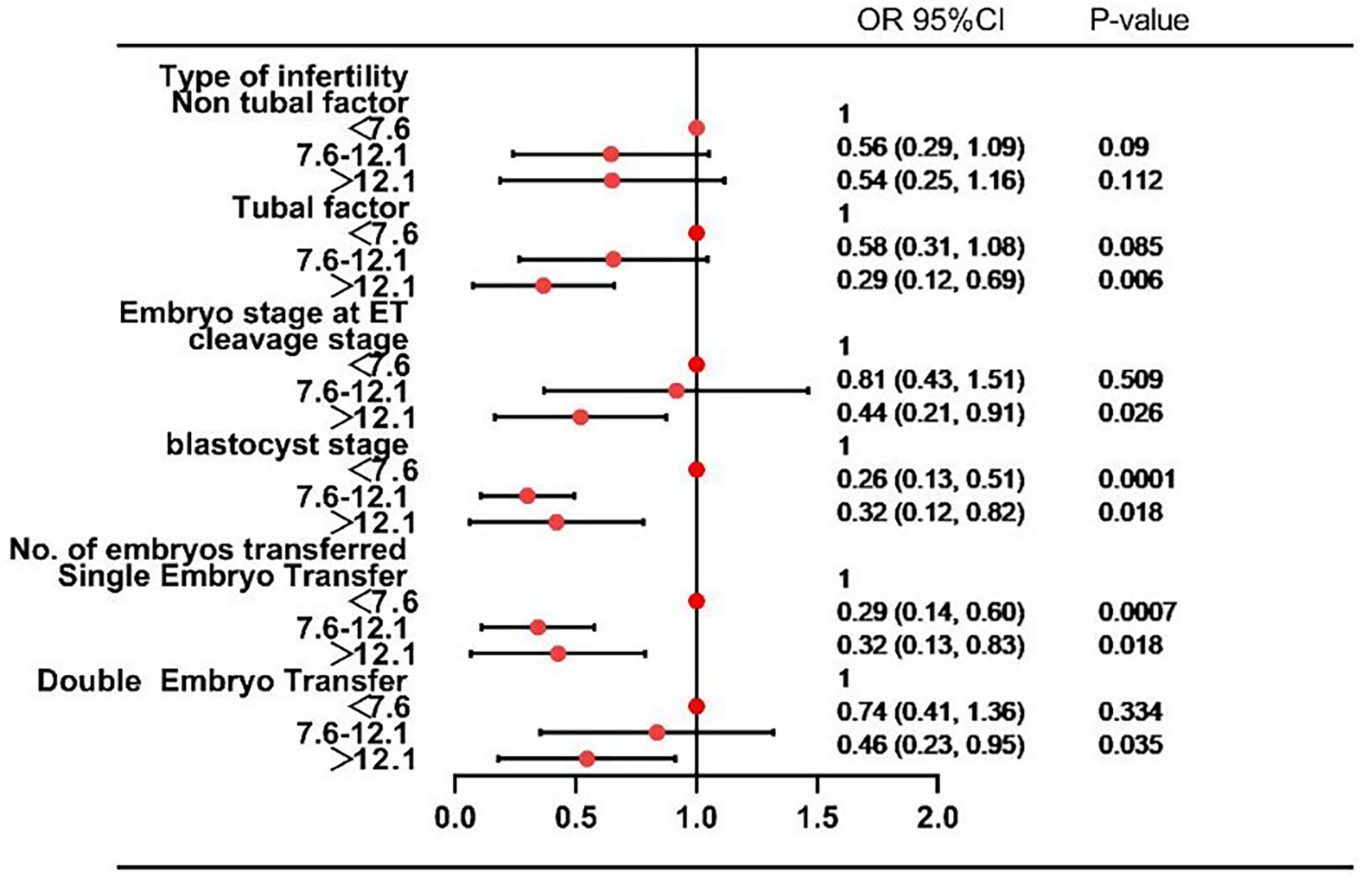
Forest plot of subgroup analyses analysis of EP risk factors.

After adjustment for female age, infertility duration, BMI, infertility type, year of treatment, cesarean section, previous EP, curettage of the uterine cavity, type of infertility, fresh or frozen embryo transfer, embryo stage at ET, embryo quality and number of embryos transferred, there was a nonlinear association between EMT and EP (**Figure 2**). We further applied three-piecewise linear regression model to examine the threshold effect of EMT on EP according to the smoothing plot. The log-likelihood ratio test comparing one-line linear regression model with three-piece-wise linear model was conducted (*P*=0.01). We stratified EMT into three groups based on cut-offs that seemed to possess clinical relevance: < 7.6 mm, 7.6 to 12.1 mm, >12.1mm. The segmented model was analyzed by multiple logistic regression to calculate the risk ratio, 95% confidence interval, and P-value (**Table 2**). The results of subgroup analysis with pelvic fallopian tube factors, EMT, embryo transfer stage, and the number of embryos transferred as stratification variables are shown in **Figure 3**. The OR values of EMT and EP in each layer were both <1, and analysis from different angles showed that the results are stable and reliable.

**Table 2:** Threshold effect analysis of endometrial thickness on EP.

	OR 95% CI	*P*
Model I		
Linear effect	0.84 (0.78, 0.90)	<0.0001
Model II		
turning point	7.6, 12.1	
< 7.6mm	0.92 (0.52, 1.64)	0.7853
7.6-12.1mm	0.75 (0.66, 0.84)	<0.0001
> 12.1mm	1.00 (0.77, 1.31)	0.997
Logarithmic likelihood ratio test	0.010	

Model was adjusted for female age, infertility duration, BMI, infertility type, year of treatment, cesarean section, previous EP, curettage of the uterine cavity, type of infertility, fresh or frozen embryo transfer, embryo stage at ET, embryo quality, number of embryos transferred.

### Univariate and multivariate analysis of the risk factors for EP

Univariate analysis revealed that pelvic fallopian tube factors (OR = 1.45, 95% CI: 1.10-1.90, *P* =0.0076), endometrial thickness (OR = 0.86, 95% CI: 0.80-0.93, *P* < 0.0001), embryo transfer stage (OR = 0.37, 95% CI: 0.27-0.51, *P* < 0.0001) and number of embryos transferred (OR = 1.88, 95% CI: 1.38-2.58, *P <*0.0001) were associated with EP. The multivariate analysis demonstrated that factors independently associated with EP were pelvic fallopian tube factors, endometrial thickness, embryo transfer stage, and the number of embryos transferred. More specifically, patients with tubal factor were associated with an almost two-fold increased risk of EP (aOR = 2.72, 95% CI: 1.69-4.39, *P* < 0.0001). An increase of EMT by one millimeter, decreased the odds of EP by 15% (aOR=0.85, 95% CI: 0.79-0.92, *P* < 0.0001). The stratified analysis showed that an EMT between 7.6-12.1mm (aOR = 0.57, 95%CI: 0.36-0.90, *P* = 0.0153) and above > 12.1mm (aOR = 0.42, 95%CI: 0.24-0.74, P = 0.0026) was significantly associated with a reduction in the risk of EP compared to women with an EMT of <7.6mm. Furthermore, blastocyst transfer can significantly reduce the risk of EP compared to cleavage stage transfer (aOR = 0.36, 95%CI: 0.26-0.50, *P*<0.0001). Double embryos transfer was associated with a nearly two-fold risk of EP (aOR = 1.99, 95% CI: 1.44 2.75) compared to single embryo transfer (**Table 3**).

**Table 3:** Univariate analysis and multivariate analysis of the risk factors for EP.

Exposure	Crude OR (95% CI)	P-value	Adjusted OR (95% CI)	P-value
Type of infertility				
Non-tubal factor	1.0		1.0	
Tubal factor	1.45 (1.10, 1.90)	0.0076	2.72 (1.69, 4.39)	<0.0001
Endometrial thickness prior to ET (mm)				
<7.6	1.0		1.0	
7.6-12.1	0.56 (0.35, 0.87)	0.0110	0.57 (0.36, 0.90)	0.0153
>12.1	0.41 (0.23, 0.72)	0.0018	0.42 (0.24, 0.74)	0.0026
Embryo transfer				
cleavage stage	1.0		1.0	
blastocyst stage	0.37 (0.27, 0.51)	<0.0001	0.36 (0.26, 0.50)	<0.0001
No. of embryos transferred				
Single Embryo Transfer	1.0		1.0	
Double Embryo Transfer	1.88 (1.38, 2.58)	<0.0001	1.99 (1.44, 2.75)	<0.0001

### Nomogram and evaluation of prediction model of EP

**Figure 4:**
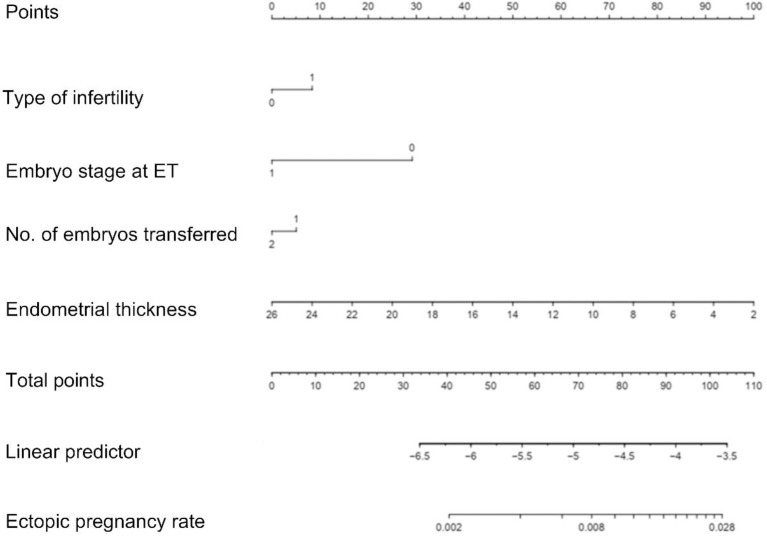
Nomogram of the clinical prediction model in the modeling group. When using a nomogram, each variable value of a patient should be determined by the corresponding point on the shaft. Applying a vertical line up to the score axis will result in the variable score. According to scores obtained for all the variables on the total score shaft, the position of the total score can be determined. Applying a vertical line down to the predicted EP rate line will give the EP probability of a patient; For illustration, a total score of 110 in the nomogram predicts a >2.8% probability of EP.

**Figure 5:**
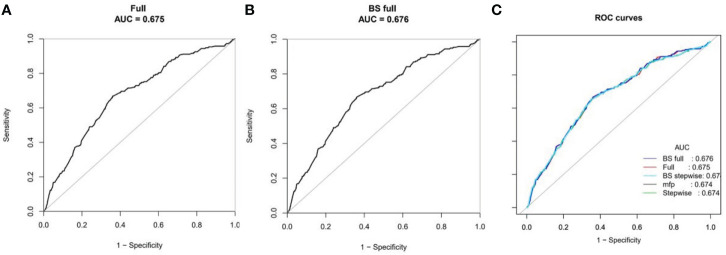
ROC curves for the accuracy of the EP nomogram **(A)**. We established three prediction models, including the full model, stepwise and MFP models. We selected the simplest full model with relatively good prediction performance to construct the nomogram to ensure clinical practicability. Bootstrap resampling validation (times = 500) confirmed the prediction performance stability of the nomogram **(B)**. The ROC curves for all models are shown in **(C)**.

Risk predictors were selected using the multivariate logistic regression model. The pelvic fallopian tube factors, EMT, the stage of embryo transfer, and number of embryos transferred were set as independent variables to predict the incidence of ectopic pregnancy. A nomogram prediction model that incorporated these significant prognostic factors was established (**Figure 4**). AUC was used to evaluate the prediction performance of the model (**Figure 5**). The risk prediction model of EP was logit P = - 1.84842 + 0.35684× (tubal factor = 1) - 1.24549 × (blastocyst = 1) - 0.21597 × (number of embryos transferred = 2) - 0.17821 × (endometrial thickness) and gave an AUC = 0.68. The good threshold (Yoden index at maximum; sensitivity + maximum specificity cutoff value) has a sensitivity of 0.67 and a specificity of 0.64, which results in a model with a moderate predictive ability (**Figure 5A**). The nomination model was internally verified by the self-sampling method (bootstrap sampling resampling times=500) and gave an AUC=0.68, sensitivity of 0.65 at the best threshold (0.46), and specificity of 0.65 (**Figure 5B**). After internal verification we could show that the AUC is basically unchanged and that the model is relatively stable (**Figure 5C**).

## Discussion

This large retrospective cohort study of 12,766 embryo transfer cycles has found that a thin EMT is an independent risk factor for EP after IVF. In addition, this study established a predictive model for the incidence of EP for the first time to provide advice for each specific patient to evaluate the incidence of EP before embryo transfer. Finally, this study found that in addition to EMT, tubal factors, the number of embryos transferred and embryo stage were significantly correlated with the incidence of EP.

One of the important findings of this study is that there is a nonlinear correlation between EMT and EP rate. When EMT ≥ 7.6mm, the incidence of EP decreased significantly. We used a three-stage linear regression model to test the threshold effect of endometrial thickness on EP rate. Compared with endometrial thickness < 7.6mm, the EMT value of endometrial thickness between 7.6~12.1mm and > 12.1mm significantly reduced the risk of ectopic pregnancy. A recent systematic review suggests that thin endometrium (< 8mm) is associated with adverse pregnancy outcomes, including early miscarriage and ectopic pregnancy (8). In another study that assessed the predictive value of EMT on the day of HCG injection for clinical outcomes in fresh cycles, the researchers found that EMT > 8mm significantly reduced the incidence of EP and that EMT was an independent variable for predicting EP (16). Another investigation suggested that women with EMT < 9mm had a four-fold higher risk of developing EP than women with EMT > 12mm (9). Although the definition of thin EMT varies according to different studies, the trend of the effect of EMT on EP rate is similar: with the increase of EMT, the incidence of EP decreases significantly. The mechanism of increased EP caused by thin endometrium is not clear currently. More literature supports that ovulation induction can lead to increased uterine peristaltic waves (17), mainly in the cervix-fundus direction (80% of 90%) (18). Enhanced peristaltic waves can squeeze embryos transferred into the uterine cavity to the outside of the uterine cavity (19). While increased EMT is a marker for increased fundus-to-cervix uterine peristalsis (9). Therefore, the thick EMT is less likely to cause the embryo to be overstocked outside the uterine cavity. In addition, the embryo transferred into the thin endometrium is closer to the uterine basilar artery, the embryo is exposed to hyperoxia, and the hypoxia environment in the fallopian tube is more suitable for embryo development and implantation, so ectopic pregnancy is more likely to occur (20).

The results of this study suggest that tubal factors are associated with a 1.7-fold increase in EP risk. The tubal factors included fallopian tube obstruction, hydrosalpinx, previous salpingectomy and salpingitis. These lesions of the fallopian tubes impair their function. Previous findings demonstrated that propulsion of the embryo from the fallopian tube to the uterus is fundamentally dependent on the well-orchestrated interaction between tubal ciliary beats, smooth muscle contractions, and the flow of tubal secretions (7). There is growing evidence that changes in the microenvironment of the oviduct such as sex steroid hormones, prostacyclin, prostaglandins, cAMP, nitric oxide (NO), and other factors might damage the ciliary activity and the smooth muscle motility, which may consequently result in a tubal ectopic pregnancy (21, 22) as the embryo cannot move to the uterine cavity on time. It has been hypothesized that redundant oxidative stress may induce smooth muscle weakness and retard oviductal ciliary beating, followed by a tubal ectopic pregnancy.

Although one previous investigation show no difference in EP rate between cleavage embryo transfer and blastocyst transfer (23), conversely, most studies demonstrated that the EP risk of blastocyst stage embryo transfer is lower than that of cleavage stage embryo transfer (6, 24, 25), which is consistent with our results. Compared with cleavage stage transfer, blastocyst transfer is closer to the physiological state, and the development of endometrium and blastocyst tends to be synchronized. This shortens the time interval for embryo transfer to remain in the uterine cavity before implantation. Blastocysts are larger than cleavage embryos, which should theoretically make it harder for blastocysts to migrate elsewhere (25). Like the published literatures (6, 11), our results also show that there is a strong correlation between the number of embryos transferred and the risk of EP, and the risk of EP in double embryo transfer is almost twice as high as that in single embryo transfer. In order to reduce the incidence of ectopic pregnancy, single embryo transfer should be considered.

One of the main findings of our study is that we have constructed a novel prediction nomogram by using a multivariate logistic regression analysis method. The clinical prediction model probably has a good discrimination ability and is likely to be applicable in clinical practice with regard to prioritizing single blastocyst transfer and considering cycle cancellation. It has the potential to predict EP after IVF using data that are easily available in routine clinical practice and may guide physicians to select personalized treatment options, as well as helping patients to obtain a better insight into their future outcomes.

Although our center performed quality control of EMT measurements, there is still some subjective variation in their results. Due to the limitation of our electronic medical records, we could not analyze the information regarding patients’ smoking history. However, according to published data, the prevalence of smoking is very low in Chinese women. Therefore, it is reasonable to hypothesize that without adjusting for smoking history, the result may not be skewed significantly (26). This study does not take into account the impact of endometriosis on the incidence of EP. This may have an impact on the final findings. In addition, this study is retrospective, so there may be some unknown confounding factors, and the data were from a single medical center. In future studies, a multi-center randomized controlled trial is needed to accommodate more patients and to generate robust data that are representative for a larger patient cohort.

In summary, our findings indicate that EMT is inversely proportional to the risk of EP. In either fresh or frozen-thawed ET cycles with an endometrial thickness ≥ 7.6 mm, single blastocyst transfer may be a better choice in order to decrease the incidence of EP in IVF treatment. The proposed nomogram has the potential to provide a new means in routine clinical practice for early prediction and recognition of EP.

## Data availability statement

The raw data supporting the conclusions of this article will be made available by the authors, without undue reservation.

## Ethics statement

The studies involving human participants were reviewed and approved by Medical Ethics Committee of the Third Affiliated Hospital of Zhengzhou University. Written informed consent for participation was not required for this study in accordance with the national legislation and the institutional requirements.

## Author contributions

JL, XY, and YG contributed to the design of the study. JL, HK, MZ, XML, and SW screened and selected articles and performed the data extraction and risk of bias assessment. HK and MZ performed the statistical analyses. JL, XY, JZ, YL, and YG interpreted data. HK and XYL wrote the manuscript. JL and YG contributed to the critical revision of the article. All authors contributed to the article and approved the submitted version.

## Acknowledgments

We would like to thank Markus Montag (ilabcomm GmbH, St. Augustin, Germany) for the valuable comments on the manuscript during the internal revision stage. We thank all of the participants and the staff of the Reproductive Center at the Third Affiliated Hospital of Zhengzhou University.

## Conflict of interest

The authors declare that the research was conducted in the absence of any commercial or financial relationships that could be construed as a potential conflict of interest.

## Publisher’s note

All claims expressed in this article are solely those of the authors and do not necessarily represent those of their affiliated organizations, or those of the publisher, the editors and the reviewers. Any product that may be evaluated in this article, or claim that may be made by its manufacturer, is not guaranteed or endorsed by the publisher.
